# Expression profile and prognostic value of NNMT in patients with pancreatic cancer

**DOI:** 10.18632/oncotarget.7891

**Published:** 2016-03-03

**Authors:** Yong Xu, Ping Liu, Dong-Hui Zheng, Nan Wu, Lun Zhu, Changying Xing, Jin Zhu

**Affiliations:** ^1^ Key Laboratory of Antibody Technique of Ministry of Health, Nanjing Medical University, Nanjing, China; ^2^ Department of Pathology, Shiyan Taihe Hospital, Hubei University of Medicine, Shiyan City, China; ^3^ Department of Nephrology, the Affiliated Huai'an Hospital of Xuzhou Medical College, Huai'an, China; ^4^ Department of Pathology, Jinling Hospital, Nanjing, China; ^5^ Department of Pathology, the Affiliated Huai'an Hospital of Xuzhou Medical College, Huai'an, China; ^6^ Department of Nephrology, The First Affiliated Hospital of Nanjing Medical University, Nanjing, China; ^7^ Huadong Medical Institute of Biotechniques, Nanjing, China

**Keywords:** pancreatic cancer, nicotinamide N-methyltransferase, clinicopathological features, overall survival

## Abstract

The elevation of Nicotinamide N-methyltransferase (NNMT) has been reported in pancreatic cancer tissues and cell lines, but its clinical and prognostic implications remain controversial. This study aimed at investigating the expression of NNMT in pancreatic benign and malignant tissues and the prognostic value of NNMT in pancreatic cancer. The expression of NNMT in tissue specimens of 28 chronic pancreatitis patients and 178 pancreatic cancer patients were assayed with immunohistochemistry on tissue microarray. The NNMT expression levels of pancreatic patients were correlated with their clinicopathological characteristics. The influences of NNMT expression and patients' clinicopathological characteristics on overall survival (OS) were analyzed. The percentage of NNMT high expression (NNMT^h^) in pancreatic cancer (55.6%) was significantly higher than those in chronic pancreatitis (21.4%) and paracancerous tissues (14.8%) (*p* < 0.001). NNMT^h^ tends to significantly correlate with unfavorable clinicopathological features such as age > 60 years old (*p* = 0.014), tumor diameter > 4 cm (*p* < 0.001), TNM stage III or IV (*p* < 0.001) and poor tumor differentiation (*p* = 0.004). The median OS of patients with NNMT^h^ and NNMT^l^ were 7.0 months (95% CI: 5.275–8.725) and 11.5 months (95% CI: 9.759–13.241) respectively (*p* = 0.005). On multivariate analysis, NNMT^l^ (hazards ratio [HR]: 0.399; 95% CI: 0.284–0.560; *p* < 0.001), absence of neurological involvement (HR: 0.651; 95% CI: 0.421–0.947; *p* = 0.041), TNM stage I or II (HR: 0.506; 95% CI: 0.299–0.719; *p* = 0.015) and well tumor differentiation (HR: 0.592; 95% CI: 0.319–0.894; *p* = 0.044) were significant favorable prognostic factors of OS. In conclusion, NNMT is upregulated in pancreatic cancer, correlates with unfavorable clinicopathological features and may serve as an independent prognosticator of patients' survival.

## INTRODUCTION

Pancreatic cancer remains one of the deadliest cancers worldwide, with the 5-year survival rate being only 7% [1, 2] and the median over survival (OS) only 6 months [3]. Due to lack of effective means for early diagnosis, most patients are diagnosed at an advanced stage that is no longer suitable for curative surgical resection. Therefore, there remains an urgent need to seek potential diagnostic biomarkers and therapeutic targets of pancreatic cancer.

Nicotinamide N-methyltransferase (NNMT) is the only known enzyme in the human body that converts nicotinamide into 1-methylnicotinamide (NMN) [4] and is involved in the biotranformation of many drugs and xenobiotics [5]. Growing evidence shows that NNMT is aberrantly expressed in and is associated with the malignancy degree of many cancers such as bladder cancer [6], lung cancer [7] colorectal cancer [8], gastric cancer [9] and hepatocellular carcinoma [10]. As for NNMT expression in pancreatic cancer, previous studies have shown that NNMT is upregulated in pancreatic cancer tissue and cell lines as well as in the pancreatic juice obtained from pancreatic cancer patients [11–13]. However, there are some controversies regarding the role of NNMT in pancreatic cancer. Bi HC, *et al.* [14] revealed that the sizes of xenograft tumors formed by PANC-1 cells (a human pancreatic cancer cell line) were inversely correlated with the levels of NNMT expression, whereas Yu T, et, al. reported that NNMT silencing and overexpression reduced and enhanced the malignancy of PANC-1 cells respectively [15]. In a small number of pancreatic cancer patients (*n* = 22), no significant association was found between the patients' overall survival and NNMT expression in the cancer specimens [14]. Therefore, the role of NNMT expression level in pancreatic cancer and its clinical significance remain elusive.

In this study, we assayed the expression levels of NNMT in tissues of chronic pancreatitis, pancreatic cancer and paracancerous specimen, and then investigated the relationship between NNMT expression level and patients' clinicopathological characteristics as well as patients' OS.

## RESULTS

### NNMT expression in chronic pancreatitis, pancreatic cancer and paracancerous tissues

**Figure 1:**
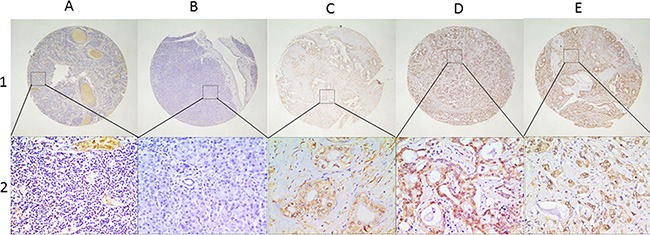
Representative presentation of NNMT protein expression in benign and malignant pancreatic tissues on tissue microarray sections by IHC Row 1 and 2 are NNMT staining observed at low (×40; bar = 500 μm) and high (×400; bar = 50 μm) magnification respectively. Column (**A**) Chronic pancreatitis with low expression of NNMT; Column (**B**) Paracancerous tissue with low NNMT expression; Column (**C–E**) show high NNMT expression in pancreatic cancer with well, moderate and poor differentiation respectively. IHC: immunohistochemistry; NNMT: Nicotinamide N-methyltransferase.

At last, a total of 178 patients with pancreatic cancer and 28 patients with chronic pancreatitis were included. Tissue samples of these patients were obtained from the Department of Pathology, Jinling Hospital (Nanjing, China), including 28 chronic pancreatitis, 178 pancreatic cancer tissues and 61 paracancerous tissues. Immunohistochemistry was performed to determine NNMT expression in these 267 samples. Representative immunohistochemically stained sections of chronic pancreatitis, paracancerous tissues and pancreatic cancer are shown in Figure 1.

Using the X-tile software program for TMA data analysis (http://www.tissuearray.org/rimmlab), we identified 110 as the significant cutoff point in terms of low and high expression of NNMT in all tissue samples, i.e., score 0–109 was considered as low expression (NNMT^l^) whereas 110–300 as high expression (NNMT^h^).

As showed in Table 1, the percentage of pancreatic cancer samples with NNMT^h^ was 55.6% (99/178), significantly higher than that of paracancerous tissues (21.4%) and chronic pancreatitis (14.8%) (*p* < 0.001), indicating that in general, NNMT is upregulated in pancreatic cancer.

**Table 1:** NNMT gene expression in pancreatic benign and malignant tissues

Tissues	*n*	NNMT^h^ (%)	Pearson χ^2^	*P*
Chronic pancreatitis	28	6 (21.4)	11.317	0.001^†^
Paracancerous tissues	61	9 (14.8)	30.629	< 0.001*
Pancreatic cancer	178	99 (55.6)	26.134	< 0.001^#^

**NNMTh:** NNMT high expression

† Chronic pancreatitis versus pancreatic cancer;

* Paracancerous tissues versus pancreatic cancer

# Among the three groups.

### Association of NNMT expression with clinicopathological characteristics in patients with pancreatic cancer

Table 2 lists the correlation between NNMT^h^ and the clinicopathological parameters of pancreatic cancer patients. As shown in this table, NNMT^h^ was significantly correlated with older age (*p* = 0.014), larger tumor size (*p* < 0.001), more advanced TNM stage (*p* < 0.001), moderate-to-poor differentiation (*p* = 0.004), and higher CA19-9 level (*p* = 0.005). These results indicate that NNMT^h^ tends to correlate with unfavorable clinicopathological features in pancreatic cancer patients compared with NNMT^l^.

**Table 2:** Association of NNMT expression with clinicopathological characteristics in patients with pancreatic cancer

Patients' characteristics		NNMT^h^ (%)	Pearson χ^2^	*P*
Total	178	99 (55.6)		
Age (years)	≤ 60 (*n* = 83)	38 (45.8)	6.094	0.014
	> 60 (*n* = 95)	61 (64.2)		
Gender	Male (*n* = 104)	57 (54.8)	0.067	0.796
	Female (*n* = 74)	42 (56.8)		
Diameter (cm)	< 4 (*n* = 107)	48 (44.9)	12.578	< 0.001
	≥ 4 (*n* = 71)	51 (71.8)		
Neurological involvement	Absent (*n* = 99)	57 (57.6)	0.346	0.556
	Present (*n* = 79)	42 (53.2)		
TNM stage	I or II (*n* = 123)	56 (45.5)	16.417	< 0.001
	III or IV (*n* = 55)	43 (78.2)		
T	T1 or T2 (*n* = 94)	52 (55.3)	0.007	0.932
	T3 or T4 (*n* = 84)	47 (56.0)		
N	N0 (*n* = 126)	59 (46.8)	13.508	< 0.001
	N1 (*n* = 52)	40 (76.9)		
M	M0 (*n* = 140)	67 (47.9)	16.001	< 0.001
	M1 (*n* = 38)	32 (84.2)		
Differentiation	Well (*n* = 17)	3 (17.6)	10.997	0.004
	Moderate (*n* = 103)	61 (59.2)		
	Poor (*n* = 58)	35 (60.3)		
Preoperative CEA (ng/ml)	≤ 5 (*n* = 106)	59 (55.7)	0.000	0.989
	> 5 (*n* = 72)	40 (55.6)		
Preoperative CA19–9 (U/mL)	≤ 34 (*n* = 74)	32 (43.2)	7.857	0.005
	> 34 (*n* = 104)	67 (64.4)		

**NNMTh:** NNMT high expression

CEA: carcinoembryonic antigen.

### The prognostic value of NNMT expression in pancreatic cancer

**Figure 2:**
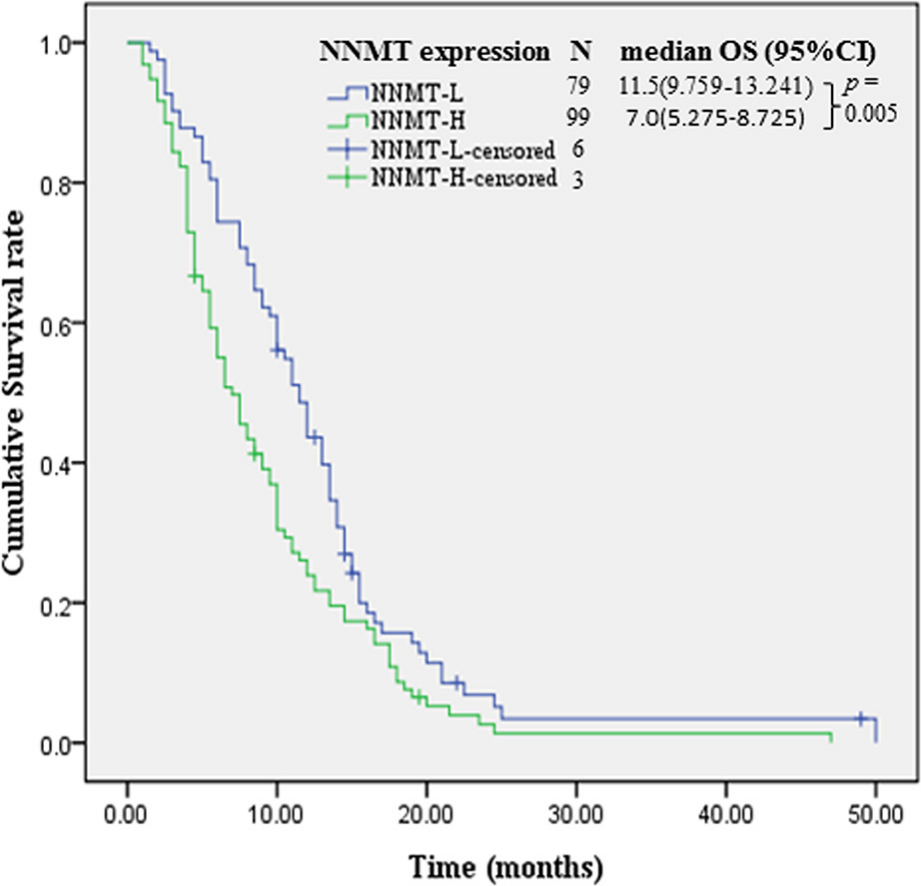
Kaplan-Meier survival curves of patients by NNMT expression levels NNMT: Nicotinamide N-methyltransferase. NNMT^h^: NNMT high expression; NNMT^l^: NNMT low expression.

By the last follow-up, only 9 patients were still alive. The median OS of patients with NNMT^h^ and NNMT^l^ were 7.0 months (95% CI: 5.275–8.725) and 11.5 months (95% CI: 9.759–13.241) respectively (*p* = 0.005) (Table 3 and Figure 2). Besides NNMT expression level, other factors that impact the median OS on univariate analyses include age (*p* = 0.011), tumor diameter (*p* = 0.027), neurological involvement (*p* = 0.033), TNM stage (*p* = 0.001), distance metastasis (*p* < 0.001) and tumor differentiation (*p* = 0.021) (Table 3).

**Table 3:** Univariate and multivariate survival analyses by clinicopathological characteristics and NNMT expression in patients with pancreatic cancer

		Univariate analysis		Multivariate analysis	
		mOS (95% CI), months	*P* value	Hazard ratio (95% CI)	*P* value
Total	178	9.0 (6.761–11.239)			
Age (years)	≤ 60 (*n* = 83)	11.0 (9.896–12.104)	0.011	0.870 (0.632–1.197)	0.392
	> 60 (*n* = 95)	6.5 (5.555–7.445)		1	
Gender	Male (*n* = 104)	9.0 (6.671–11.239)	0.312		
	Female (*n* = 74)	9.0 (7.679–10.321)			
Diameter (cm)	< 4 (*n* = 107)	11.2 (10.019–12.412)	0.027	0.759 (0.525–1.116)	0.103
	≥ 4 (*n* = 71)	7.4 (6.169–9.007)		1	
Neurological Involvement	Absent (*n* = 99)	10.7 (9.343–12.002)	0.033	0.651 (0.421–0.947)	0.041
	Present (*n* = 79)	7.2 (6.024–9.121)		1	
TNM stage	I or II (*n* = 123)	10.0 (8.470–11.530)	0.001	0.506 (0.299–0.719)	0.015
	III or IV (*n* = 55)	7.5 (5.156–9.844)		1	
T	T1 or T2 (*n* = 94)	10.6 (8.829–11.771)	0.47		
	T3 or T4 (*n* = 84)	8.5 (6.742–10.258)			
N	N0 (*n* = 126)	10.4 (8.508–11.792)	0.127		
	N1 (*n* = 52)	8.0 (6.459–9.541)			
M	M0 (*n* = 140)	9.7 (8.733–11.267)	< 0.001	0.706 (0.485–1.056)	0.092
	M1 (*n* = 38)	6.8 (4.558–8.442)		1	
Differentiation	Well (*n* = 27)	11.4 (8.966–14.434)	0.021	0.592 (0.319–0.894)	0.044
	Moderate (*n* = 69)	9.5 (7.447–11.553)		0.832 (0.611–1.121)	
	Poor (*n* = 82)	7.2 (5.601-8.689)		1	
CEA^#^ (ng/ml)	≤ 5 (*n* = 106)	9.8 (6.977–13.023)	0.373		
	> 5 (*n* = 72)	8.5 (6.681–10.319)			
CA19–9^#^ (U/mL)	≤ 34 (*n* = 34)	10.0 (8.314–11.686)	0.326		
	> 34 (*n* = 144)	7.5 (5.385–9.615)			
NNMT expression	NNMT^h^ (*n* = 99)	7.0 (5.275–8.725)	0.005	1	< 0.001
	NNMT^l^ (*n* = 79)	11.5 (9.759–13.241)		0.399 (0.284–0.560)	

# Preoperative

mOS: median overall survival; CEA: carcinoembryonic antigen.

On multivariate analysis, NNMT^l^ (hazards ratio [HR]: 0.399; 95% CI: 0.284–0.560; *p* < 0.001), absence of neurological involvement (HR: 0.651; 95% CI: 0.421–0.947; *p* = 0.041), TNM stage I or II (HR: 0.506; 95% CI: 0.299–0.719; *p* = 0.015) and well tumor differentiation (HR: 0.592; 95% CI: 0.319–0.894; *p* = 0.044) were significant favorable prognostic factors of OS compared with NNMT^h^, presence of neurological involvement, TNM stage III or IV and poor differentiation, respectively (Table 3).

## DISCUSSION

In this study, we showed that the percentage of NNMT^h^ in pancreatic cancer was significantly higher than those in pancreatic benign tissues, in line with the previous findings that NNMT is elevated in pancreatic cancer [11,12]. Moreover, we for the first time revealed that NNMT^h^ tends to correlate with unfavorable clinicopathological features in pancreatic cancer patients and that NNMT^h^ is an independent unfavorable prognosticator of OS, suggesting an oncogenic role of NNMT in this cancer.

Previous studies have reported an increased incidence of pancreatic cancer in patients with a history of chronic pancreatitis [16]. Our study shows that the percentage of NNMT^h^ in pancreatic cancer was significant higher than that in chronic pancreatitis (55.6% vs 21.4%, *p* < 0.001), indicating that NNMT expression level has the potential to distinguish pancreatic benign from malignant lesions.

To date, there are only very few reports [14, 15] suggesting the possible roles of NNMT in pancreatic cancer and the results are controversial. Bi HC, *et, al.* [14] reported that the sizes of xenograft tumors were inversely correlated with NNMT expression, suggesting an oncosuppressive role of NNMT in pancreatic cancer. Yu T, et, al. [15], on the contrary, reported that NNMT silencing and overexpression reduced and enhanced the malignancy of pancreatic cancer cells respectively, suggesting an oncogenic role. Our results showed that NNMT^h^ tends to correlate with unfavorable clinicopathological features in pancreatic cancer patients, in support of its oncogenic role, although the underlying mechanisms remain elusive. Bi HC, *et, al.* [14] had investigated the prognostic value of NNMT expression level in a small number of pancreatic cancer patients (*n* = 22) but failed to find any positive result, possibly due to the small cohort size. Our study for the first time revealed that compared with NNMT^h^, NNMT^l^ is an independent and significant favorable prognosticator of patients with pancreatic cancer.

As we know, the dismal prognosis of pancreatic cancer is at least partially attributable to the lack of effective and convenient means of early diagnosis. During the past decades, many potential biomarkers have been tested [17] but only CA 19–9 shows great promise [18, 19]. Yet, CA 19–9 is not a satisfactory biomarker because it is not specific enough [20] and about 10% of pancreatic cancer patients present negative CA 19–9 value even in advanced stages of the disease [21]. Therefore, there remains an urgent need to develop novel biomarkers. Our study adds a new member to the molecular candidates holding potentials to be developed as diagnostic and prognostic biomarkers of pancreatic cancer.

Although patients with TNM stage III and IV pancreatic cancer are unsuitable for surgical resection in mainstream opinions [22], some stage III or IV patients in our hospital indeed received surgical treatment for palliative purposes upon the request of patients and after the careful evaluation by the institutional expert team. So, there were 55 patients with stage III or IV pancreatic cancer in our study whose participation enabled us to investigate the expression and prognostic value of NNMT in this subset of patients. One limitation of our study is that we did not set foot in the mechanisms underlying NNMT elevation and the mechanisms underlying its prognostic value in pancreatic cancer, which should be addressed by future studies.

In conclusion, NNMT is upregulated in pancreatic cancer, correlates with unfavorable clinicopathological features and holds the potential to be developed as aprognosticator of patients' survival. The results of our study require further validation in a larger patient cohort and the mechanisms underlying these results merit further investigation to explore the possibilities of NNMT as a therapeutic target of pancreatic cancer.

## MATERIALS AND METHODS

### Human tissue specimens and patient clinical information

This study was approved by the Human Research Ethics Committee of Jinling Hospital (Nanjing, China). Treatment-naive patients with pancreatic cancer who received pancreatectomy as the initial treatment at Jinling Hospital (Nanjing, China) from Jan 1st, 2010 to Dec 31st, 2013 were included in this study. The diagnoses of pancreatic cancer were confirmed by postoperative pathological results. Patients with chronic pancreatitis confirmed by fine needle aspiration biopsy during the same period were also included.

Clinical characteristics of cancer patients were extracted from their medical record, including age, gender, tumor diameter, neurological involvement, TNM stage, differentiation, preoperative serum carcinoembryonic antigen (CEA) and carbohydrate antigen 19–9 (CA19– 9) levels. Patients with pancreatic cancer were followed up and the OS were calculated from the date of surgical treatment to the date of death or last follow-up.

### Immunohistochemistry analysis (IHC) on paraffin embedded tissue

The tissue microarray block was cut into 4-μm sections and immunohistochemical staining was performed as previously described [23]. Briefly, the sections were first deparaffinized and hydrated. After antigen retrieval with 0.01 M citrate buffer pH 6.0 and microwave heat induction, the sections were treated with 3% hydrogen peroxide for 10 min to block the endogenous peroxidase activity. NNMT was detected by mouse monoclonal anti-human NNMT antibody (dilution 1:150) (Novus Biologicals, USA, NBP2-00537). After secondary antibody staining, diaminobenzidine (DAB) was used as the chromogen for 3 min, and then the nuclei were counterstained with hematoxylin. Staining results were evaluated independently by two pathologists without prior knowledge of clinicopathologic data.

The expression of NNMT was scored using the semi-quantitative H-score method, which takes into account both the staining intensity and the percentage of cells at that intensity [24]. For each of the samples, the staining intensity was scored as 0 (no staining), 1+ (weak staining), 2+ (moderate staining), or 3+ (intense staining). Then the percentage of cells stained at the respective intensity was determined and multiplied by the intensity score to yield an intensity percentage score. The final staining scores were then calculated from the sum of the four intensity percentage scores. Therefore, the staining score had a minimum value of 0 (no staining) and a maximum value of 300 (100% of cells with 3+ staining intensity).

### Statistical analysis

The continuous NNMT expression data from IHC were converted into dichotic data (low vs high) using specific cutoff values, which were selected to be significant in terms of OS using the X-tile software program (The Rimm Lab at Yale University; http://www.tissuearray.org/rimmlab) [25].

Student *t* test and Pearson χ2 test were used to determine the statistical significance of differences between comparison groups. Median OS was calculated using the Kaplan-Meier method and compared by the log-rank test. Variables with *P*-value < 0.20 on univariate analyses were included in multivariate analysis (Cox proportional hazards model). *P*-value less than 0.05 was considered statistically significant. All statistical analyses were performed using SPSS 20.0 statistical software package (SPSS Inc., Chicago, IL).
